# Modern Environmental Health Hazards: A Public Health Issue of Increasing Significance in Africa

**DOI:** 10.1289/ehp.0800126

**Published:** 2009-01-29

**Authors:** Onyemaechi C. Nweke, William H. Sanders III

**Affiliations:** 1 Office of Policy, Economics, and Innovation and; 2 Office of Children’s Health Protection and Environmental Education, U.S. Environmental Protection Agency, Washington, DC, USA

**Keywords:** Africa, environmental health, hazards

## Abstract

**Objectives:**

Traditional hazards such as poor sanitation currently account for most of Africa’s environmentally related disease burden. However, with rapid development absent appropriate safeguards for environment and health, modern environmental health hazards (MEHHs) may emerge as critical contributors to the continent’s disease burden. We review recent evidence of human exposure to and health effects from MEHHs, and their occurrence in environmental media and consumer products. Our purpose is to highlight the growing significance of these hazards as African countries experience urbanization, industrial growth, and development.

**Data sources:**

We reviewed published epidemiologic, exposure, and environmental studies of chemical agents such as heavy metals and pesticides.

**Data synthesis:**

The body of evidence demonstrates ongoing environmental releases of MEHHs and human exposures sometimes at toxicologically relevant levels. Several sources of MEHHs in environmental media have been identified, including natural resource mining and processing and automobile exhaust. Biomonitoring studies provided direct evidence of human exposure to metals such as mercury and lead and pesticides such as *p*,*p′*-dichlorodiphenyltrichloroethane (DDT) and organophosphates. Land and water resource pollution and industrial air toxics are areas of significant data gaps, notwithstanding the presence of several emitting sources.

**Conclusion:**

Unmitigated MEHH releases and human exposure have implications for Africa’s disease burden. For Africans encumbered by conditions such as malnutrition that impair resilience to toxicologic challenges, the burden may be higher. A shift in public health policy toward accommodating the emerging diversity in Africa’s environmental health issues is necessary to successfully alleviate the burden of avoidable ill health and premature death for all its communities now and in the future.

According to the World Health Organization (WHO), about one-third of Africa’s disease burden is attributable to environmental hazards (Prüss-Üstün and Corvalán 2006). The major contributing risk factors to environmental disease burden in the continent are traditional environmental health hazards such as lack of access to safe water, indoor air pollution from solid fuel combustion, and lack of sanitation and hygiene. However, with notable economic growth the past decade (World Bank 2008), urbanization, and continuing industrialization, modern environmental health hazards (MEHHs) can be expected to eventually emerge and perhaps supersede traditional hazards as critical contributors to environmental disease burden in the continent. The transition to MEHHs is in progress, as evidenced by the combination of preindustrial- and industrial-era environmental health issues confronting many African communities (WHO 2002). Assuring population health and well-being in the near future, therefore, will depend not only on how well traditional hazards and risks are managed but also on the degree to which MEHHs and their potential impacts are prevented or controlled.

MEHHs are products of rapid development in the absence of health and environment safeguards, as well as the unsustainable consumption of natural resources (WHO 1997). MEHHs include, but are not limited to, water pollution from populated areas and industry, urban air pollution from automobiles, radiation hazards, land degradation, climate change, and emerging and reemerging infectious diseases (Corvalán et al. 1999). The occurrence of several MEHHs and their sources has been noted in industrialized and urbanized African communities [United Nations Environment Programme (UNEP) 2002]. Industrial pollution, in particular, is becoming highly concentrated in growing urban areas, and as a result, the continent’s pollution intensity (pollution generated per unit of production output) is among the highest in the world [United Nations Industrial Development Organization (UNIDO) 2004].

In this review, we highlight the increasing significance of MEHHs as a public health issue in Africa using several lines of evidence, in no particular order: the presence of development-related activities (e.g., industry) that are capable of generating these hazards; evidence of the occurrence of these types of hazards in environmental media as a result of development activities; evidence of human exposure to some of these hazards; and evidence of adverse effects among African populations. We weave these lines of evidence through chemical- specific examples such as toxics from industrial activity, air pollution from automobiles, and pesticides from modern agricultural practices. Our focus on chemical hazards is solely based on the relative preponderance of data for these types of hazards and is therefore very narrow in terms of the scope of possible MEHHs that are products of development. Accordingly, this is not a comprehensive review of all MEHHs in Africa. However, it more than suffices for our goals for this review, which are to demonstrate that MEHHs are increasing in significance as an environmental health concern, and also to emphasize the need for these hazards to occupy a priority spot in Africa’s public health and policy agenda.

## Examples of MEHHs in Africa

### Mercury

Mercury, in several forms, is a known neurotoxin that causes cognitive deficits at low exposure levels and severe neurologic effects at very high levels of exposure. Hg also exerts toxic effects on other organ systems, notably, the kidneys and the cardio vascular and immune systems. A detailed review of the range of toxic effects associated with exposure to different forms of Hg has been published previously [Agency for Toxic Substances and Disease Registry (ATSDR) 1999b]. An important source of direct human exposure to high levels of Hg in Africa is artisanal gold mining and processing. Hg exposures from artisanal gold mining are mostly to the vaporized metallic form (Savornin et al. 2007). Specifically, the highest exposure to vaporized elemental Hg occurs during burning to separate gold from the gold–Hg amalgam. According to a study of the process among artisanal gold miners in Tanzania and Zimbabwe, up to 1.46 g of Hg is lost to the environment per gram of recovered gold, and 70–80% of these releases are to the atmosphere (van Straaten 2000). The release of Hg during processing occurs within the breathing zone of the worker, who is typically not equipped with personal protective equipment (PPE). Individuals in close contact with workers, such as children under the care of mothers who work as processors, are also at risk of exposure. Dermal exposure may also occur from contact with Hg-contaminated mining waste or during amalgam handling.

Exposure to Hg in artisanal mining is a particularly important public health issue because of the demographics of the industry: Women and children are engaged in gold mining, and their involvement spans all possible activities, including those associated with the highest risks of Hg exposure. In the Kedougou region of Senegal, the involvement of women and children ranges from ore extraction to burning amalgam, and in the Tenkoto region, the process of amalgamation is carried out by women, within the vicinity of their young children (Savornin et al. 2007). In Gaoua, Burkina Faso, the mining and sale of gold have traditionally been a female-only activity (Hentschel et al. 2003). According to Hentschel et al. (2003), the proportion of the African artisanal workforce composed of women and children can range from approximately 5% in South Africa (*n* = 500) to 50% in Mali (*n* > 100,000). They also estimate that > 3,000 children are directly employed in artisanal mining in Tanzania. Children engaged in artisanal mining cut across a wide range of ages. In a 1998 survey summarized by Gueye (2001), about 59% of 500 children working at artisanal mining sites in Burkina Faso were ≤ 12 years of age.

Children are vulnerable to the toxic effects of Hg, particularly during the earliest stages of neuro development. The neurotoxic effects of Hg during development are well documented for methylmercury (MeHg) and discussed extensively elsewhere [National Research Council (NRC) 2000]. Unfortunately, a comparable volume of data on the developmental effects of metallic Hg exposure is not available. Despite the relative paucity of data on the effects of metallic Hg on children, similarities with MeHg in ways that predict toxicity suggest that the same target systems may be affected. For example, absorption of inhaled metallic Hg in blood is very high, and 70–80% is retained; its *in vivo* distribution is similar to that of organic Hg; and it is readily transferred through the placenta and blood–brain barrier because of its lipophilicity (ATSDR 1999b). Clinical symptoms of neurotoxicity have been noted in children exposed to metallic Hg via artisanal mining. Bose-O’Reilly et al. (2008) reported statistically significant differences in the prevalence of neurotoxic symptoms, such as excessive salivation, ataxia of gait, and abnormal reflexes in exposed children relative to controls (*n* = 166; blood Hg range, < 0.20 to 100.8 μg/L; urine Hg range, < 0.20 to 941.9 μg/L). Performance on tests such as pencil tapping and matchbox tests, which evaluate neuromotor function, was also statistically significantly poorer in exposed children (Bose-O’Reilly et al. 2008).

Data on Hg exposures among African artisanal miners are very limited. However, a few recent studies provide insight into the extent of exposure among these miners relative to the non miners in their communities [see Supplemental Material, Appendix C, Table 1 (available online at http://www.ehponline.org/members/2009/0800126/suppl.pdf)]. Overall, these studies demonstrate elevated body burdens of Hg in exposed populations such as miners, workers involved in ore processing, and even children residing in mining communities. In one study of gold miners in Kadoma, Zimbabwe, the highest Hg exposures occurred in amalgam burners relative to other workers (Boese-O’Reilly et al. 2004). Median blood Hg concentration among study participants was 5.62 ppb; the equivalent blood Hg concentration for the U.S. Environmental Protection Agency’s (EPA) current reference dose for MeHg exposure is 5.8 ppb. Further analysis of the Kadoma study participants identified elemental Hg as the primary form to which the study group was exposed (Boese-O’Reilly et al. 2004). A similar study of an artisanal mining community in Gyapa, Ghana, showed a median blood Hg concentration of 10.4 ppb and a maximum concentration of 44.8 ppb (Rambaud et al. 2003). The study sample predominantly comprised persons who indicated working in artisanal gold mining (83%).

Significant body burdens of Hg have been reported in individuals exposed through sources other than occupational mining. Harada et al. (2001) reported high total Hg concentrations in the hair of African females known to habitually use soaps containing high concentrations of inorganic Hg; the highest level of total Hg in hair (900 ppm) was observed in a fisher woman who used Hg-containing soap. When they excluded users of European-made Hg-containing soaps, the highest total hair Hg concentration recorded was 6.2 ppm. Total Hg levels in the European-made Hg-containing soaps were about 3 orders of magnitude higher than the levels in locally made soaps. Levels of total Hg among study participants did not show any particular trends with duration of soap use (possibly due to poor recall or variations in use) or being a resident of a gold-mining area, urban area, or fishing village (Harada et al. 2001). Fish consumption among persons with high exposure was not reported, although the authors indicated that an earlier survey of the base population strongly suggests that Hg exposure was unlikely through the food chain. Developmental neurotoxicity after *in utero* exposure may occur at maternal hair concentrations > 10 ppm in women exposed to organic Hg (ATSDR 1999b). The prevalence of African women who habitually use Hg-containing cosmetic products is not known; however, surveys in certain populations suggest that the use of these cosmetic products is quite variable, ranging from 10% in a study of Senegalese women (Mahe et al. 2003) to 47% in a population of male and female Nigerian traders (Adebajo 2002). The current availability of Hg-containing cosmetics in the African market is not known.

Consumption of fish contaminated with MeHg is also a potential source of human exposure to Hg in Africa. Surveys of fish caught in African lakes, rivers, and streams show large variation in terms of the levels of MeHg in fish relative to recommended limits. Mercury levels in 20 species of commercial fish caught from the Gulf of Guinea in Ghana were lower than international levels of concern; the highest Hg level was lower than the WHO’s recommended limit of 0.5 μg/g wet weight (Voegborlo et al. 2004). Similar results were found in fish from the hydroelectric reservoirs in Tanzania (Ikingura and Akagi 2003). In contrast, sampling conducted in waters potentially affected by gold mining show exceedances of the WHO limit. In the Kadoma-Chakari region of Zimbabwe, Billaud et al. (2004) observed exceedances of the WHO limit in some carnivorous and one omnivorous fish species. Appleton et al. (2004) also reported exceedances of the WHO limit in fishes caught in the immediate areas of gold-mining activities at Rwamagasa, Tanzania. These data suggest that, in addition to direct occupational exposures to metallic Hg, residents of gold-mining communities may be exposed to MeHg in their diet via ingestion of fish caught from local water bodies contaminated by mining activities.

### Lead

Lead is a naturally occurring heavy metal. Pb exposure is associated with several adverse effects across different population subgroups. Most studied and understood is the effect of Pb on neurodevelopment and, in particular, its negative effects on intelligence quotient (IQ) (Lanphear et al. 2005) and behavior (Chen et al. 2007; Needleman et al. 1996). More recently, Pb has been associated with other adverse health impacts, such as increased risks of attention-deficit hyperactivity disorder in children (Braun et al. 2006) and cardiovascular mortality in adults (Schober et al. 2006).

The sources of Pb with the highest risk of exposure in Africa include deteriorated house paint, leaded gasoline, mining operations, polluted water, and contaminated foods and cosmetics (Nriagu et al. 1996). Other identified sources in Africa include contaminated dust and soil (Liggans and Nriagu 1998); contaminated ceramics, crayons, pencils, and piped water (Okonkwo and Maribe 2004); Pb-acid battery manufacturing and disposal (Kimani 2005); and Pb-containing paint used in housing (Montgomery and Mathee 2005). Consumer products, in particular, are emerging as potential sources of exposure to high concentrations of Pb in Africa [see Supplemental Material, Appendix C, Table 2 (http://www.ehponline.org/members/2009/0800126/suppl.pdf)].

Until recently, leaded gasoline was the key source of widespread population exposure in urbanized parts of Africa. With its elimination from all of sub-Saharan Africa in 2007 [Partnership for Clean Fuels and Vehicles (PCFV) 2007], sources such as Pb in paint and residual Pb in soil and dust from the legacy of leaded gasoline will increase in relevance as sources of widespread human exposure on the continent. It is also important to note that high but confined population exposures to Pb in communities such as Kabwe, Zambia, as a result of large emissions from sources such as natural resource mining (Blacksmith Institute 2006) remain a significant public health problem.

The Pb content of paint merits further elaboration because recent studies of the Pb content of paint used in African households and schools, or of paint that is available on the African market, strongly indicate that Africa has not benefited from the vast experience of more industrialized countries in dealing with the problem of Pb paint hazards. Montgomery and Mathee (2005) recently found that 20% of homes sampled in the South African town of Johannesburg (*n* = 239) had at least one Pb-based paint sample (Pb content ≥ 5,000 ppm). In the southwestern city of Ibadan, Nigeria, almost all (96%) samples (*n* = 25) of glossy paints manufactured and sold locally had Pb content ≥ 600 ppm (Adebamowo et al. 2007). It is noteworthy that the median concentration of Pb measured in the Nigerian study was 15,800 ppm, with a maximum concentration of 50,000 ppm. The U.S. Consumer Product Safety Commission (CPSC 1977) recommended that the Pb content of paint should not exceed 600 ppm based on a finding of “unreasonable risk of lead poisoning in children associated with lead content of over 0.06 percent in paints and coatings to which children have access.”

Major reasons for the continued high Pb content of paint include the nonexistence of standards for Pb in paint in African countries. In South Africa, where the environmental health agenda is relatively advanced, legislation to restrict the use of Pb in paint was still in progress as of 2007 (Mathee et al. 2007). Paint containing Pb is an important source of Pb exposure for children in the home environment because of direct consumption of paint chips or dust and soil contaminated with deteriorated paint. Increased levels of Pb in surrounding dust and soil are both positively associated with elevated blood Pb (Lanphear et al. 1996, 1998).

Several Pb exposure studies have been conducted among children and adults in urban, rural, and occupational settings using blood Pb as the metric of exposure [see Supplemental Material, Appendix C, Table 1 (http://www.ehponline.org/members/2009/0800126/suppl.pdf)]. These studies are representative of exposures primarily in rural and occupational settings and, in one case report (Mathee et al. 2003), of exposure to Pb paint through pica. We included data on blood Pb levels in adults (Adeniyi and Anetor 1999; Robins et al. 1997) because childhood exposure to Pb can occur during pregnancy. In addition, children of occupationally exposed parents may be exposed to Pb from contaminated work clothes. We have largely excluded urban data in anticipation that the phased removal of Pb in gasoline should result in a shift in the distribution of blood Pb levels in urban populations, such that blood Pb distribution parameters from these past studies are unlikely to be representative of the current distribution of blood Pb in these populations. This expectation is supported by a blood Pb survey of inner-city children in South Africa 6 years after the introduction of unleaded gasoline, showing a statistically significant reduction in mean blood Pb levels over this time period (Mathee et al. 2006).

Other sources of Pb exposure highlighted in the literature include occupational exposure, potential transfers of Pb from contaminated work clothes to family members, and residence in a mining community. Widespread community exposure to Pb as a result of proximity to Pb mining has been demonstrated in the United States (ATSDR 2002; Murgueytio et al. 1998). In a study of two mining communities in South Africa, von Schirnding et al. (2003) identified residence in these communities and the transfer of Pb dust from contaminated work clothes as potentially important risk factors for elevated blood Pb levels among sampled residents.

### Pesticides

Pesticide exposure among African populations is a public health concern for many reasons, including but not limited to the widespread and poorly regulated use of pesticides and the increased likelihood of pesticide exposures attributable to lack of training, lack of access to information, or high frequency of pesticide use given climatic conditions that favor the proliferation of pests and weeds. Pesticides are used for pest control in several environments such as in the home and in various forms of agriculture. However, more opportunities for high-dose pesticide exposures and widespread releases to the environment may exist for pesticide use in agriculture.

The footprints of pesticide use in Africa are evident in food and environmental media. For example, pesticides used in agriculture have been detected in streams, rivers, and sediments that are located within the vicinity of farms in different regions of Africa (Dalvie et al. 2003; Ntow 2001; Schulz 2001a, 2001b). Pesticides have also been detected in groundwater used for drinking water. For example, Dalvie et al. (2003) reported that endosulfan levels (mean ± SD) in ground water samples taken from agricultural areas across locations in South Africa ranged from 0 ± 0 to 0.83 ± 0.99 μg/L, regularly exceeding “the European drinking water standard of 0.1 μg/L.” The number of water samples taken at each location ranged from 1 to 22 (Dalvie et al. 2003). In the studies of Dalvie et al. (2003), Ntow (2001), and Schulz (2001a, 2001b), typical pesticides detected in environmental media included organochlorines such as endosulfan, lindane, and heptachlor epoxide. *p*,*p′*- Dichlorodiphenyldichloro-ethylene (DDE), a breakdown product of the pesticide *p*,*p′*-dichlorodiphenyltrichloroethane (DDT), has also been detected in environmental media. The frequent reporting of organochlorines in the environment does not necessarily imply that organochlorine pesticides are the most used in Africa, but it may reflect their persistence in the environment and scientific interest in this class of compounds. In fact, carbamates, organophosphates, and pyrethroids, which are less persistent in the environment, are also widely used for agricultural purposes.

Pesticides may contaminate food items during application (Amoah et al. 2006; Ntow 2001) or through bioaccumulation in the food chain. When persistent pesticides such as organochlorines are used in food production, the chances of bioconcentration by plants and organisms in the environment and bioaccumulation up the food chain are increased. Although some African countries may have residue limits for pesticides in food, lack of access to country-level information is an impediment to identifying such countries and to understanding how these limits are enforced to assure consumer safety. Obsolete pesticides stocks also are a serious public health issue for Africa; these pesticides contaminate soil, water, air, and food sources and pose serious health threats to Africa’s rural and urban populations (UNEP 2006).

Human exposure to pesticides used in agriculture may be highest among farmworkers and their families. In addition to experiencing more frequent exposures to a wide array of pesticides, farmworkers and their families are likely to be exposed to high doses by multiple routes. For example, pesticides used in agriculture can be tracked into the home on clothes and shoes (Lu et al. 2000; Solomon and Mott 1998). Such tracking is less likely when appropriate PPE, such as coveralls, is used and protective measures such as bathing or hand washing and removing clothes worn on farms are employed. However, recent surveys of African farmers suggest that only a small percentage of farmers consistently use appropriate PPE (Clarke et al. 1997; Matthews et al. 2003; Ntow et al. 2006). Cost is frequently cited as a significant impediment to the appropriate use of PPEs in these surveys. Other factors that limit the use of PPEs are the hot and humid climatic conditions, nonprovision of equipment by employers, and the belief that PPE is not needed or not effective (Clarke et al. 1997). Pesticide exposure may also occur via dermal contact with treated crops (Lu et al. 2000).

Pesticide use in the home environment may result in exposure as a result of contact with residues after application, storage, or disposal of pesticides. Pesticides such as pyrethroids and DDT (in some countries) are sprayed indoors to reduce mosquitoes and control malaria transmission and may contaminate house dust. House dust is a known medium through which exposure to environmental contaminants occurs, particularly if the contaminants are persistent in the environment. Unlike pyrethroids, DDT is slowly biodegraded and therefore persistent in the environment (Turusov et al. 2002).

There is biomonitoring evidence of human exposure to pesticides in Africa. Inhibition of acetylcholinesterase (a biomarker of exposure and effect) has been measured in Kenyan farmworkers who used organophosphates (Ohayo-Mitoko et al. 2000). In another study, Kinyamu et al. (1998) found that breast milk samples (*n* = 216) taken from nonfarmer urban Kenyan mothers up to 4 weeks postpartum also had detectable levels of nine organochlorine pesticides. The most detected organochlorine pesticide in that study was DDT (and its metabolite DDE), although others, such as dieldrin and lindane, were also detected. The ranges of measured concentrations of organochlorine pesticides in breast milk (milk fat basis) were as follows: DDT, 0.002–2.58 mg/kg; DDE, 0.003–4.818 mg/kg; dieldrin, 0.004–0.273 mg/kg; and lindane, 0.002–0.134 mg/kg. Based on intake calculations, Kinyamu et al. (1998) suggested that infants whose mothers had the highest levels of DDT (generally presented as the sum of the parent compound and its metabolites) and dieldrin would exceed the WHO/Food and Agricultural Organization (FAO) acceptable daily intakes for these organochlorine pesticides (DDT, 20 mg/kg; dieldrin, 0.1 mg/kg) at the time the study was conducted. An earlier study of exposure to DDT among mothers residing in areas of Kwazulu, South Africa, also reported detectable levels of DDT and its metabolites [DDE and *p*,*p′*-dichloro diphenyl-dichloroethane (DDD)] and isomer (σ-DDT) in breast milk samples (Bouwman et al. 1990). DDT was used for malaria control in certain parts of the study area, and mothers residing in these parts of the study area had mean breast milk DDT levels (15.83 mg/kg in milk fat) that were statistically significantly higher than those of mothers residing in parts of the study area where DDT was not used (0.69 mg/kg in milk fat). Both DDE and the organochlorine pesticide contaminant/residue hexachlorobenzene have also been detected in breast milk (mean DDE, 490 μg/kg fat) and blood (mean DDE, 380 μg/kg) samples taken from residents of a farming community in Ghana (Ntow 2001). Collectively, these studies document historical and ongoing pesticide exposure in individuals most likely exposed occupationally through agriculture and in those exposed through routes other than an occupation in agriculture.

Very few health effect studies of farm-worker populations have directly assessed the adverse effects associated with single or multiple pesticide use and exposure among African populations, and no studies have examined the toxicologic consequences of interactions resulting from cumulative exposures to several pesticides in these populations. Most of the available studies on farmworker populations rely on self-reported information to diagnose adverse effects and therefore may not provide the most objective data. Nonetheless, these studies have reported adverse effects such as neurotoxicity associated with organo-phosphate exposure (Ohayo-Mitoko et al. 2000) and birth defects in an agricultural community (Heeren et al. 2003). Despite the general lack of epidemiologic data relating specific pesticide exposures to adverse health effects among African populations, the classes of pesticides that have historic or current widespread use in Africa (e.g., organophosphates, organochlorines, carbamates, pyrethroids) have been extensively toxicologically evaluated for a range of adverse effects as part of the process of registration for purposes of use, and are known to induce a range of adverse health effects. A toxic effect central to all four classes of pesticides is neurotoxicity (NRC 1993; U.S. EPA 2006). The endocrine-disrupting properties of pesticides are also an emerging area of research (Cohn et al. 2007; Hodges et al. 2000; Perry et al. 2006; Ulrich et al. 2000).

### Air Toxics

Outdoor air pollution in particular has emerged as an issue the last few decades, particularly in Africa’s urban centers because of increased rates of urbanization and industrialization (UNEP 2002). An important contributor to air pollution in Africa’s cities is automobile emissions. In recent air sampling conducted in Dares Salaam, Tanzania, measured sulfur dioxide (SO_2_) levels (hourly mean SO_2_ concentration range, 127–1,385 μg/m^3^) in parts of the city (eight locations) exceeded the 1987 WHO daily hourly average guideline for SO_2_ (350 μg/m^3^), with the exception of one site (Jackson 2005). High levels of SO_2_ and suspended particulate matter (PM) were both correlated with traffic flow. Other air toxics typically emitted from automobile exhausts include volatile organic compounds and nitrogen oxides. Quantitative data on the impact of automobiles on outdoor air quality are very limited because air quality studies are not routinely conducted for most African cities. However, some of the publicly available and most recent data show a range of air pollution data for PM, SO_2_, and nitrogen dioxide across different studies and African communities and, more important, highlight pollutant concentration ranges that suggest some communities or populations are exposed to levels of air pollution that exceed recommended limits [see Supplemental Material, Appendix C, Table 3 (http://www.ehponline.org/members/2009/0800126/suppl.pdf)].

Industrial activity is also an important contributor to outdoor air pollution in parts of Africa, and the type of air quality issues in an affected area is typically sector specific, aside from the effects of long-range transport. Many African countries, for example, are heavily invested in natural resource mining (Coakley and Mobbs 2002). As a result, the impact of this industrial sector on ambient air quality can be profound in communities with mining and processing activities. A 2002 environmental assessment for the World Bank’s Copper Environment Project in the Zambia copper belt reported that Cu smelter plants released an estimated 300,000–700,000 tons of SO_2_ annually (ZCCM Investment Holdings Plc 2002). The report concluded that exposure to SO_2_ and PM was the “primary environmental health issue” in certain communities within the region. The “South Africa Environment Outlook” report (Department of Environmental Affairs and Tourism 2006) also noted that some local communities in South Africa face a high risk of exposure to airborne asbestos fibers from abandoned, unrehabilitated, and/or disturbed mines and randomly discarded asbestos fiber dumps. An earlier study similarly attributed widespread asbestos contamination of communities located around mills and mines in South Africa to asbestos mining activity (Marchand 1991). Exposure to contaminant-laden fugitive dust in communities located in mining areas is not unusual. Community exposure to air pollution resulting from proximity to mining activities has been noted for asbestos and Pb in Libby, Montana (Horton et al. 2006), and Herculaneum, Missouri (ATSDR 2002), respectively, both in the United States.

Besides the risk of exposure to the mined mineral, a primary source of concern with air pollution from natural resource mining is exposure to other cooccurring minerals such as uranium, radium, vanadium, Cu, and arsenic that may also contaminate fugitive dusts. In the Obuasi region of Ghana, for example, gold extraction from arsenopyrite ore results in the release of airborne PM that includes large concentrations of As (Mead 2005).

Several other types of industry with potentially significant contributions to outdoor air pollution other than natural resource mining are operational in Africa; these include pulp and paper mills, chloralkali industry, cement industry, organic chemical industries, battery production, metallurgical industry, energy production, and oil and gas production and refining (Shannon 2004) [see Supplemental Material, Appendix C, Table 4 (http://www.ehponline.org/members/2009/0800126/suppl.pdf)]. Highly industrialized regions, such as the eastern Transvaal Highveld region in South Africa, are noted for their high concentration of industries such as petrochemical plants; as of 1990, coal-fired power plants in that region generated 80% of South Africa’s electrical energy (Zwi et al. 1990).

Indoor air pollution is also an important source of exposure to air toxics in many African homes. A primary source of indoor air pollution is the burning of domestic fuels used in the home (e.g., biomass, charcoal, wood, coal). Although traditionally an issue in rural environments, it is an emerging issue in low-income and rapidly urbanizing environments. Other sources of indoor air pollution include pesticides used in the home and cigarette smoking [see Supplemental Material, Appendix A (http://www.ehponline.org/members/2009/0800126/suppl.pdf)].

Some epidemiologic studies have been conducted to evaluate the health effects (particularly respiratory effects) of indoor and outdoor air pollution exposure on African populations. Wichmann and Voyi (2005) provided an extensive review of most of these studies, which are limited to South African populations. According to the authors, “the studies provide some evidence of associations with a range of serious and common health problems,” although they also noted that the studies they reviewed have limited validity and precision because of significant problems with systematic and random errors.

Air toxics are associated with a wide array of health effects, and health standards for several of these pollutants have been developed to protect for these adverse effects, particularly in countries with a more developed environmental health agenda, such as the United States. Exposure to PM, for example, has been associated with increased mortality and morbidity from cardiovascular and respiratory diseases (U.S. EPA 2005). Both formaldehyde and acetaldehyde are classified as carcinogenic and possibly carcinogenic to humans, respectively [International Agency for Research on Cancer (IARC) 1999, 2006]. Formaldehyde is also a respiratory tract irritant (ATSDR 1999a). Some polycyclic aromatic hydrocarbons are carcinogenic in humans and more recently have been linked to non cancer effects such as poor birth outcomes (e.g., low birth weight) in certain study populations (Boy et al. 2002; Perera et al. 2003). Some health effects are also associated with exposure to smoke from solid fuel combustion. Health effects associated with smoke in the literature include acute lower respiratory infections (ALRI), chronic obstructive pulmonary disease (COPD), and asthma (Bruce et al. 2000). Increased risk of ALRI among young children, in particular, has been documented in observational studies, some of which were conducted in African populations (Smith et al. 2000). The relationship between smoke and adverse respiratory outcomes have been discussed extensively by Smith et al. (2000) and Desai et al. (2004).

### Water Contaminants

Sources of drinking water in Africa are ground-water, surface waters (e.g., streams, lakes, and rivers), and sometimes rainfall. Dependence on each source varies with factors such as availability and proximity of a type of source, degree of urbanization, and level of infrastructure development. The extent to which surface water and groundwater quality in Africa is affected by development activities has not been extensively studied or reported. However, a few published reports provide some insight into potential sources of pollution and the likely extent of the problem. The “Africa Environment Outlook” report (UNEP 2002) identifies ground water pollution from nitrates, phosphates, and chemical residues used in Africa as a concern, particularly in countries that are highly dependent on underground aquifers for drinking water. In another report focused on the Zambezi River Basin of South Africa, Chenje (2000) specifically noted several point and nonpoint sources of pollution that affect both surface water and groundwater quality, including sewage-treatment facilities, pulp and paper mills, fertilizer factories, abattoirs, textile and cloth manufacturing entities, mining activities, agriculture, and chemical industries. The author further estimated an annual release of about 93,000 metric tons of industrial waste into the Zambezi River from these activities. Like many major rivers in Africa, the Zambezi River is an important water resource. In 2005, a collaborative effort between the University of Nairobi and UNEP (UON/UNEP 2005) collected voluntary wastewater effluent data from industries that discharge into the Nairobi River Basin in Kenya; some industries in one division reported effluent concentrations for chromium (2,400 mg/L), nickel (59 mg/L), Cu (580 mg/L), and Pb (120 mg/L) that were much higher than recommended local effluent guidelines. Specifically, the report indicated that reported concentrations for Ni, Cu, and Pb were 60, 600, and 120 times higher than the local recommended effluent guidelines, respectively.

### Domestic and Hazardous Waste

Waste is an unavoidable product of development-related activities such as industrialization and urbanization. We discuss waste in two categories: municipal waste from domestic sources and hazardous waste from industrial activity. Municipal waste management in most of Africa’s cities is a significant environmental problem (Achankeng 2003). In South Africa, the estimate of total domestic waste generated from all its provinces was 42.2 million m^3^ per annum (Mpumalanga Department of Agriculture, Conservation and Environment 2003). In Africa’s cities, municipal solid waste collection rates can be as low as 20%, thus resulting in illegal dumping (Achankeng 2003). Municipal waste is typically disposed of in open dumps (with the exception of South Africa) or sanitary landfills (Johannessen and Boyer 1999). With increased economic growth and urbanization, the nature of domestic waste in many African communities is evolving to include MEHHs such as cadmium and Pb from used batteries.

There is inadequate information about the generation of hazardous wastes in Africa. However, the Department of Environmental Affairs and Tourism (2006) provided some insight into the amounts of such waste generated by major industrial sectors in South Africa. According to the report, the total hazardous waste generated in the 1997/1998 financial year by the four largest industrial sectors (nonmetallurgical manufacturing, metallurgical and metal industries, service industries, and mining) exceeded 418 million tons, of which about 90% came from mining in the form of tailing dams, slag dams, and rock dumps. The Department of Environmental Affairs and Tourism (2006) also noted the presence of other wastes such as radioactive wastes, asbestos waste, and power generation waste; power generation waste, in the form of waste ash from nine coal-fired power stations in 2006, was estimated to be 33 million tons per annum. Environmentally sound management of wastes such as heavy metals from disposed batteries, electronic wastes, and wastes from medical sources, as well as commercial and industrial activity, is an identified area of need in Africa (WHO/UNEP 2008a).

Where attempts have been made to manage waste, efforts have sometimes been inadequate because of the use of nonprotective technologies (unlined landfills) and the location of landfills near residential quarters or in wetlands and other areas with seasonally high water tables (Alimba et al. 2006). As various industrial sectors, such as agriculture and chemical manufacturing, continue to experience unprecedented growth and evolution, and lifestyles become modernized, the lack of or inadequacies in municipal and hazardous waste treatment plans and facilities can be expected to result in more profound impacts of development-related activities on environmental media (e.g., water, land resources), with potentially grave consequences for human health.

### MEHHs in Consumer Products

The discussion of potential sources of human exposure to MEHHs in Africa is incomplete without mention of the contribution of consumer products. Certain consumer goods designed to improve lifestyles may contain toxic substances to which humans can be exposed. One example is Pb in paint. As discussed above, concentrations of Pb in paint sold and manufactured in African countries remain very high despite the preponderance of knowledge on the health effects of Pb and the contribution of paint to human exposure.

Asbestos cement roofing sheets are another example of a consumer product that can result in human exposure to a hazardous agent. Asbestos has been historically used in roof construction in Africa. In the Niger Delta region of Nigeria, asbestos roofing is the material of choice to counter the corrosive action of acid rain that is prevalent in the region (Clark et al. 1999). In isolation, asbestos roofing sheets do not pose a hazard; bulk material deterioration and the consequential release of fibers that can be inhaled or ingested is what creates the hazard. Exposure to asbestos fibers may lead to lung cancer, mesothelioma, or asbestosis. Cigarette smoking is also known to interact synergistically with asbestos to increase the risk of lung cancer (ATSDR 2001).

There are no data on the prevalence of asbestos roofs in African countries or actual asbestos hazards from the use of asbestos in roofing. However, one study suggested that lack of maintenance and poor handling of asbestos materials, both of which are likely to yield asbestos hazards, may be common in African communities where this product has been used. In a survey of the South African suburb of Soweto, 17% of homes with asbestos roofs had roofs that were in some state of disrepair (Mathee et al. 2000). In addition, 62% of these homes lacked ceilings that could serve as a barrier to indoor exposure to fibers. Mishandling of asbestos sheets was also reported; some sawing of asbestos sheets was reported in 6% of the asbestos-roofed homes, and 46% of this subset of respondents recalled creating dust during such work. Knowledge of asbestos as a hazard was very low among respondents. This survey was administered to mothers of 1,488 singleton newborns in 1990. The authors reported that 52% of these children resided in houses with asbestos roofing (Mathee et al. 2000).

Overall use of asbestos among African countries declined significantly the last two decades, with the exception of Zimbabwe, which reported an “apparent” total use rate > 100,000 metric tons for the years 2000 and 2003 (Virta 2006). Using 1975 as a base comparison year, Zimbabwe’s annual consumption rate increased an order of magnitude over two decades. Given the overall temporal decline in asbestos consumption among most African countries, it is reasonable to presume that the legacy of asbestos roofing is mostly confined to countries with a history of large consumption, such as Nigeria, South Africa, and Ghana. However, the extent to which country-level consumption patterns reflect the use of asbestos in roofing materials and other consumer goods, and therefore the extent of the asbestos hazard issue in the environment, remains a significant data gap.

Other consumer products such as food, drinks, traditional medicines, Hg-containing cosmetics, and food treated with pesticides or veterinary drugs also may serve as potential sources of human exposure to potentially harmful chemicals. For example, a recent survey of heavy metal levels in commonly consumed canned (*n* = 21) and noncanned (*n* = 29) beverages available for purchase in the Nigerian market indicates a high prevalence of beverages with levels of total As and total Cr that exceed the U.S. EPA’s maximum contaminant levels (MCLs) for these metals (As_MCL_ = 0.01 mg/L; Cr_MCL_ = 0.10 mg/L) (Maduabuchi et al. 2007). Levels of total As ranged from 0.002 to 0.261 mg/L, and levels of total Cr ranged from 0.01 to 0.59 mg/L in both types of beverages. According to the authors, the overall prevalence of MCL exceedances in both canned and noncanned beverages was 46% for total As and 72% for total Cr. In another study, Obi et al. (2006) evaluated herbal remedies sold in Nigeria for heavy metal content and found the following ranges of estimated adult daily intakes: iron, 11,500–123,750 μg/day; Ni, 2,750–78,000 μg/day; Cd, 550–4,750 μg/day; and Cu, 15,000–97,500 μg/day. Estimated daily intakes of these metals resulting from consumption of the herbal remedies exceeded the stated recommended tolerable upper intake levels for Ni (1,000 μg/day), Fe (4,500 μg/day), and Cu (10,000 μg/day) (NRC 2001) and the provisional tolerable weekly intake for Cd (converted to a daily intake of 70 μg/day, assuming a body weight of 70 kg) (FAO/WHO 2003).

## Conclusion

The reviewed body of evidence lends support to the perspective that Africa’s environmental health issues are complex and extend beyond the more obvious long-standing traditional hazards such as malaria, safe drinking water, and basic sanitation. The full range of MEHHs released into the African environment, and thus the levels to which its populations are exposed, is unknown. Examples of MEHHs for which we found evidence of releases in the African environment include pesticides, air toxics, and heavy metals such as Pb and Hg. Some of these MEHHs have also been found in such consumer goods as food items, household paint, and crayons. Worth noting is evidence of very high concentrations of Pb in purchase-ready household paint despite several decades of research demonstrating that Pb-based paint is associated with Pb exposure and harmful health effects in children. Also noteworthy is evidence of contamination of food sources and herbal remedies with high levels of heavy metals. Conclusive evidence of exposure to MEHHs based on body burden data was generally lacking. However, the relatively robust exposure database for Pb demonstrates elevated body burdens in African populations exposed to contaminated sources and provides suggestive evidence of ongoing exposures to MEHHs at biological levels that are associated with adverse health impacts. Studies linking human exposures to health effects were the least available and represent an important data gap.

Critical to the emergence of MEHHs as an environmental health issue is the context within which this evolution is occurring [see Supplemental Material, Appendix B (http://www.ehponline.org/members/2009/0800126/suppl.pdf)]. These emerging environmental challenges to health are occurring within the context of strained health systems (WHO/UNEP 2008a) characterized by impaired or significantly limited capacities and a population burdened with a high prevalence of other vulnerability factors such as poverty, malnutrition, and HIV/AIDS. The vulnerability status of African populations is a very important consideration because exposures and health risks from most environmental hazards are unevenly distributed, often affecting vulnerable populations most heavily (WHO/UNEP 2008b).

The issue of MEHHs in Africa has been the focus of several health protective initiatives over the years. Examples include the efforts of the PCFV initiated by UNEP to remove Pb from gasoline and and phase down sulfur from diesel products sold in Africa; the Africa Stockpiles program aimed at reducing the stockpiles of obsolete pesticides in Africa; the ongoing UN Development Programme/UNIDO-sponsored Global Mercury Project, which aims to introduce cleaner technologies for gold extraction, educate artisanal miners in the application of these methods, and introduce environmental and health monitoring programs; and the activities of the Air Pollution Information Network Africa in the areas of disseminating information on air pollution issues, collating and assessing emission sources, levels, and impacts of air pollution, and developing air quality guidelines.

Generally, these activities originate in response to concern over an MEHH issue and are therefore mostly problem specific (e.g., pesticides, air pollution, or Hg in artisanal mining). This focused approach has proved effective using the example of the Pb phaseout activities of the PCFV. Moving forward, however, what should prove even more effective in terms of reducing the impact of MEHHs on the health of African populations are environmental health actions and policies that reduce the cumulative burden of MEHHs, because a focus on one MEHH and source at a time provides improvements in overall health status of a population that are eroded by the impacts of MEHHs and sources that remain uncontrolled. As Africa’s environmental health issues become more complex and the continent’s “riskscape” under goes significant change, environmental health policy and action must adopt more comprehensive, holistic, and population-specific approaches for identifying, recognizing, and managing environmental health hazards. Resolving the long-standing issues of traditional hazards in tandem with tackling emerging MEHH issues is necessary to successfully alleviate the burden of avoidable ill health and premature death for all of Africa’s communities.
